# Dietary Approaches to Stop Hypertension: Lessons Learned From a Case Study on the Development of an mHealth Behavior Change System

**DOI:** 10.2196/mhealth.3307

**Published:** 2014-10-23

**Authors:** Devin M Mann, Lisa M Quintiliani, Shivani Reddy, Nicole R Kitos, Michael Weng

**Affiliations:** ^1^Boston UniversityDepartment of MedicineBoston, MAUnited States; ^2^Boston UniversityDepartment of General Internal MedicineBoston, MAUnited States; ^3^Boston UniversityDepartment of Preventive Medicine & EpidemiologyBoston, MAUnited States; ^4^Boston UniversityDepartment of Computer ScienceBoston, MAUnited States

**Keywords:** mHealth, chronic disease, behavior change

## Abstract

**Background:**

Evidence-based solutions for changing health behaviors exist but problems with feasibility, sustainability, and dissemination limit their impact on population-based behavior change and maintenance.

**Objective:**

Our goal was to overcome the limitations of an established behavior change program by using the inherent capabilities of smartphones and wireless sensors to develop a next generation mobile health (mHealth) intervention that has the potential to be more feasible.

**Methods:**

In response to the clinical need and the growing capabilities of smartphones, our study team decided to develop a behavioral hypertension reduction mHealth system inspired by Dietary Approaches to Stop Hypertension (DASH), a lifestyle modification program. We outline the key design and development decisions that molded the project including decisions about behavior change best practices, coaching features, platform, multimedia content, wireless devices, data security, integration of systems, rapid prototyping, usability, funding mechanisms, and how all of these issues intersect with clinical research and behavioral trials.

**Results:**

Over the 12 months, our study team faced many challenges to developing our prototype intervention. We describe 10 lessons learned that will ultimately stimulate more effective and sustainable approaches.

**Conclusions:**

The experiences presented in this case study can be used as a reference for others developing mHealth behavioral intervention development projects by highlighting the benefits and challenges facing mHealth research.

## Introduction

### Background

Cardiovascular disease remains the leading cause of morbidity and mortality in the United States [1]. Despite well-characterized drivers—diet, physical activity, obesity—and effective treatments for its key risk factors, such as hypertension, the burden continues. Several decades of research have produced successful behavior change interventions that promote a healthier approach to diet and physical activity with notable highlights in the field of hypertension. The Dietary Approaches to Stop Hypertension (DASH) and its more comprehensive successors, the PREMIER and POWER lifestyle modification programs [2-4], promote a lifestyle approach to blood pressure control that emphasizes a diet rich in fruits and vegetables with moderate portions of low-fat dairy and lean protein, along with increased physical activity and reduced sodium intake. These well-validated studies provide an ample evidence base for the effectiveness of lifestyle modification for hypertension control, but to date remain underused. Multiple factors underlie the underuse of evidence-based interventions, including the intense resources required to replicate the intervention, the substantial user burden required to participate, and the low engagement of participants over time [5,6]. Researchers and dissemination partners who attempt to promote evidenced-based strategies to large populations are quite familiar with factors limiting uptake of behavior change interventions, not just for hypertension control, but for health promotion in general.

### Clinical Problem

We imagine an “average” primary care patient named Dawn. Dawn is 35 years old, is overweight, works in a sedentary office environment, and gets little leisure-time exercise. Her primary care provider has been following Dawn’s low grade hypertension over the past three office visits (last blood pressure reading was 145/85). In the current clinical environment, comprehensive hypertension control programs based on DASH, PREMIER, and POWER are not widely available in most practices; as such, no referral to a lifestyle program is made. With the few minutes available, the provider reviews her usual diet and physical activity habits, recognizes need for improvement, perhaps prints out a “DASH hypertension control” reference booklet from her electronic medical record and hands it to Dawn with directions to read it before the next visit in 3 months.

### Potential of mHealth for Behavioral Interventions

In the current health care setting of episodic physician visits, low provider knowledge/confidence of behavioral health promotion, insufficient ancillary support services, and nearly non-existent behavioral data feedback from patients, mHealth offers a promising means of delivering effective lifestyle interventions. Mobile technologies such as smartphones and wireless sensors, along with the dynamic software that powers them, have the ability to deliver behavioral interventions in a simple, personalized, and objective manner. Below, we present our rationale as to why smartphones can be a powerful modality for delivering mHealth behavioral interventions for hypertension control. (1) Smartphones can be embedded into patients’ lives and are increasingly common. More than half (56%) of Americans report owning a smartphone, up from 46% in 2012 and 35% in 2011 [7]. They can deliver health interventions—educational and/or motivational—in the patient’s usual context. It is the patient-driven customization to their personal lifestyle that is critical. (2) They can also deliver engaging, multimedia content that goes far beyond the traditional patient education booklet described above, which is still the dominant resource in the average clinician’s repertoire. (3) Smartphones can integrate with new wireless devices that can collect patient data such as blood pressure, weight, and physical activity to drive tracking and feedback loops for discrete data and wirelessly sync with smartphones [8,9]. The automatic collection of data by these devices dramatically reduces the need for tedious data entry, allowing patients to focus their efforts on engaging with the app’s tracking and feedback systems [10]. Data are also collected objectively, compared to self-reported data, providing more accurate feedback for patients and information for the health care team. (4) Smartphones are able to deliver personalized feedback and data visualizations to facilitate a potentially more meaningful relationship with the data [11,12], a motivational concept based on several key theories of persuasion and change including the Elaboration Likelihood Model and traditional operant conditioning [13,14], and (5) utilize GPS and other mapping functionalities such as Foursquare to customize the intervention to the patients’ local environment. (6) Smartphones can embed alerts, reminders, or game-based motivational tools into mobile apps, weaving these tools into the daily fabric of patient’s lives and promoting engagement with behavior change interventions, and (7) serve as hubs for social interventions using platforms like Patients Like Me or Facebook, or social networking forums built specifically for the intervention [9].

Unfortunately, most lifestyle apps for conditions such as hypertension do not effectively leverage the evidence base or smartphones capabilities [15]. In an October 2013 brief search of the DASH-like apps available on Google Play for Android and the Apple app store for iOS, apps related to DASH were primarily information resources, providing tips about hypertension and treatment or shopping lists and recipes. While these apps can be interactive (eg, tailored shopping lists), they did not leverage the multiple communication channels afforded by smartphones to help patients follow the behavior change plan or have comprehensive self-monitoring capabilities to track weight and physical activity, which are all integral components of hypertension management.

The purpose of this case study is to describe our experience developing a behavioral blood pressure reduction mHealth system designed from the outset to leverage the unique capabilities of smartphones and wireless sensors to facilitate learning, utilization, engagement, and motivation. Throughout our description, we will highlight lessons learned with a focus on current and potential future research pathways.

## Methods

### Creation of DASH Mobile

In response to the clinical need and the growing capabilities of smartphones, our study team decided to develop a DASH-inspired behavioral hypertension reduction mHealth system. DASH was successfully migrated to an Internet version over a decade ago [16]. The Internet program has been studied and has demonstrated significant, albeit less dramatic, effects compared to the original, more intensive in-person version. However, the Internet version was limited by poor participant engagement over time and has not been disseminated widely [16]. For our DASH mobile system, one of the first decisions was to build our program from scratch. Starting with the fully developed Internet version would serve to constrain our design and limit the system’s flexibility. Therefore, the DASH Mobile development project reimagined the behavioral hypertension trial evidence using this guiding question: “How would we design an app that can deliver the underlying behavioral-based blood pressure reduction concepts using the tools of mHealth”? In retrospect, this decision represents one of the key lessons learned: creating some distance from traditional approaches to behavior change delivery formats designed for historical technological environments is critical to developing tools that can fully leverage the technology. Next, we began the process of team assembly, identifying funding, and planning our step-wise development process as will be explained in the following sections. Our goal was to design a first generation, minimally viable product that would be ready for efficacy and feasibility testing within one year. While one year may appear long compared to rapid industry prototyping lifecycles, the complexities of academic bureaucracies (institutional review boards, etc), funding mechanisms, multidisciplinary teams, academic calendars and student participation, and lack of mHealth development experience in academic medical centers may slow the development cycles.

### Assembly of the Team and Acquiring Start-Up Funding

The first step was to assemble the appropriate team [17]. The required expertise was broad including primary care, behavior change, nutrition, computer science, design, human-computer interactions, usability, videography, and informatics. Funding is a scarce resource on academic medical campuses, but multidisciplinary, technology-driven projects are more likely to be developed, evaluated, maintained, and successfully built upon with adequate start-up funding. The relatively slow funding cycle and careful ramp-up of new technologies in academia creates further drag on this type of innovation [6]. Funding early in the development cycle provides several advantages, including creation of a more interactive, industry-quality user interface (UI), more options for linking data collection devices, and a pilot study among a larger sample of participants allowing for sufficient preliminary data to drive the next steps in the research process and successfully compete for highly competitive, limited traditional grant opportunities. While funding mechanisms such as the National Institutes of Health (NIH) Small Business Innovation Research program exist to enable innovation of projects designed for the marketplace, in our experience, smaller funds with shorter and simpler application cycles are potentially better suited to early mHealth development stages. In the case of DASH mobile, our work was sponsored by two pilot grants: one from our university and another from a local technology consortium incubator (Center for Integration of Medicine and Innovative Technology).

### Approaches and Frameworks

mHealth is an exciting new tool for developing health behavior change interventions, but it is just a tool. Careful thought needs to be given to the behavioral framework that will support and ultimately drive the technology facing the patient. While many frameworks exist, there are several that warrant highlighting as important touch points for our intervention design. The first is Michie’s COM-B behavior change [18], which organizes behavior change into three components: Capability, Opportunity, and Motivation. This is a reformulation of decades of behavioral research and is a useful system for categorizing potential drivers of behavior and behavioral intervention design. Another organizing force in our development was the increasingly influential persuasion science literature. Leaning heavily on the work of Cialdini, the persuasion literature has advocated for the development of behavior change tools that take greater advantage of the peripheral route in the Elaboration Likelihood Model [13]. Messages processed through the mind’s peripheral route are filtered through heuristics that do not involve more complex central processing and are sensitive to external signals from the environment as guides to decision making. Approaches that promote behavior change by tapping into these automatic, almost reflex responses that individuals can have to stimuli could and should be combined with traditional cognitive behavior interventions. We used these complementary frameworks as a lens for the design and implementation of the DASH mobile system, which we believe will be critical to the success and ultimate efficacy of our mHealth system.

### Starting the Development Process

The first major design decision is determining the scope and key components of the mHealth intervention. Scoping decisions included the following questions and answers.

#### Would This Be a Standalone App or Would We Include a Coaching/Counseling Component?

**Figure 1:**
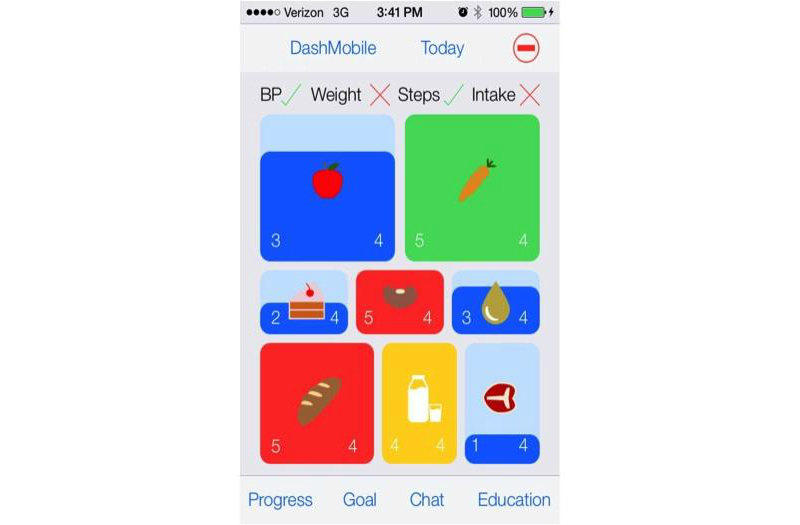
Screenshot from DASH Mobile: Self-reported DASH diet interface.

**Figure 2:**
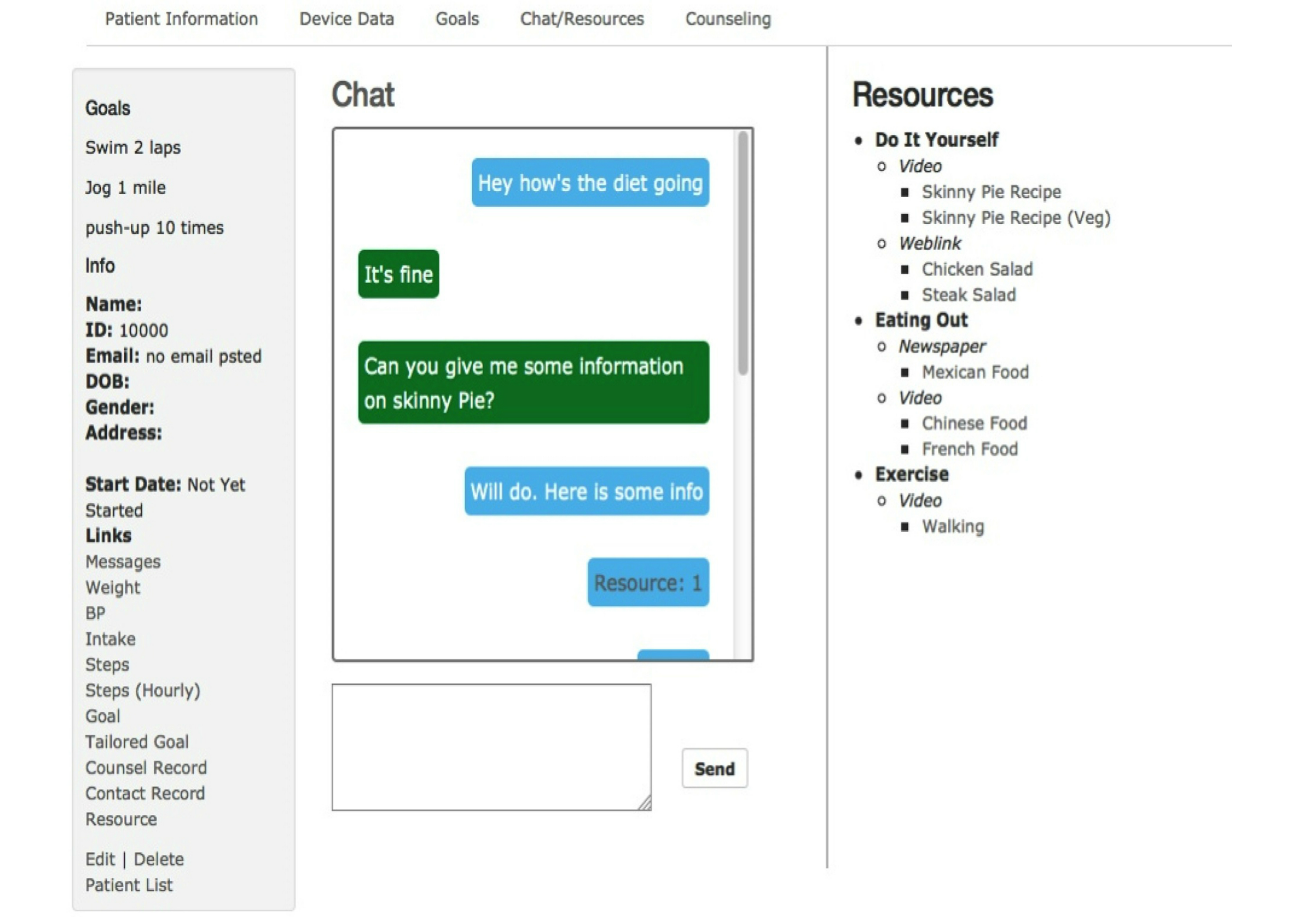
Screenshot from DASH Mobile: Prototype DASH e-coaching portal.

We ultimately decided to include a human-based mHealth coach component to our system in order to capitalize on the capabilities of a human counselor to dynamically interact with a patient during a conversation, tailoring the conversation to the issues at hand in real time [19,20]. Motivational interviewing was selected as the counseling framework because its iterative, dynamic, and flexible approach based on client feedback is particularly well suited to human interaction. Motivational interviewing is a psychologically based health behavior change counseling method suited for those ready to take action and, importantly, also for people who are ambivalent about change [21]. A counselor trained in motivational interviewing techniques aims to work with a person’s core values and beliefs, resolve the person’s natural ambivalence, and express their own internal motivations to change [21]. Interventions using motivational interviewing have demonstrated efficacy for changing lifestyle behaviors among patients with a range of diverse sociodemographic characteristics [22,23]. In addition, the use of a human coach may also be leveraged to increase sustained use with the mHealth system [24]. With these principles in mind, we decided to build a comprehensive mHealth solution that involved a patient-facing smartphone app and coach-facing Web-based portal (Figures 1 and 2). In order to preserve the potential scalability of our mHealth system, we built features into the system that allow the human coach to have both synchronous and asynchronous communication with a patient.

#### Who Would Be the mHealth Coach?

This is an important and complicated question. In the end, we wanted to avoid an mHealth coach who would be unlikely in real world settings due to misaligned skillsets and high cost (eg, physicians and nurses). However, we were also unsure how well an mHealth coach with specific training in counseling might perform. As such, we selected a Master’s level student as the mHealth coach, although we anticipate adapting this role to the level of a health coach, navigator, or community health worker using standardized training and scripts. This decision was also based on funding considerations, given that our initial pilot funding precluded hiring a professional counselor but was suited to providing training in the core concepts of motivational interviewing, education about the behavioral topics, and opportunities to practice counseling along with provision of feedback.

#### How Would the Devices Communicate?

**Figure 3:**
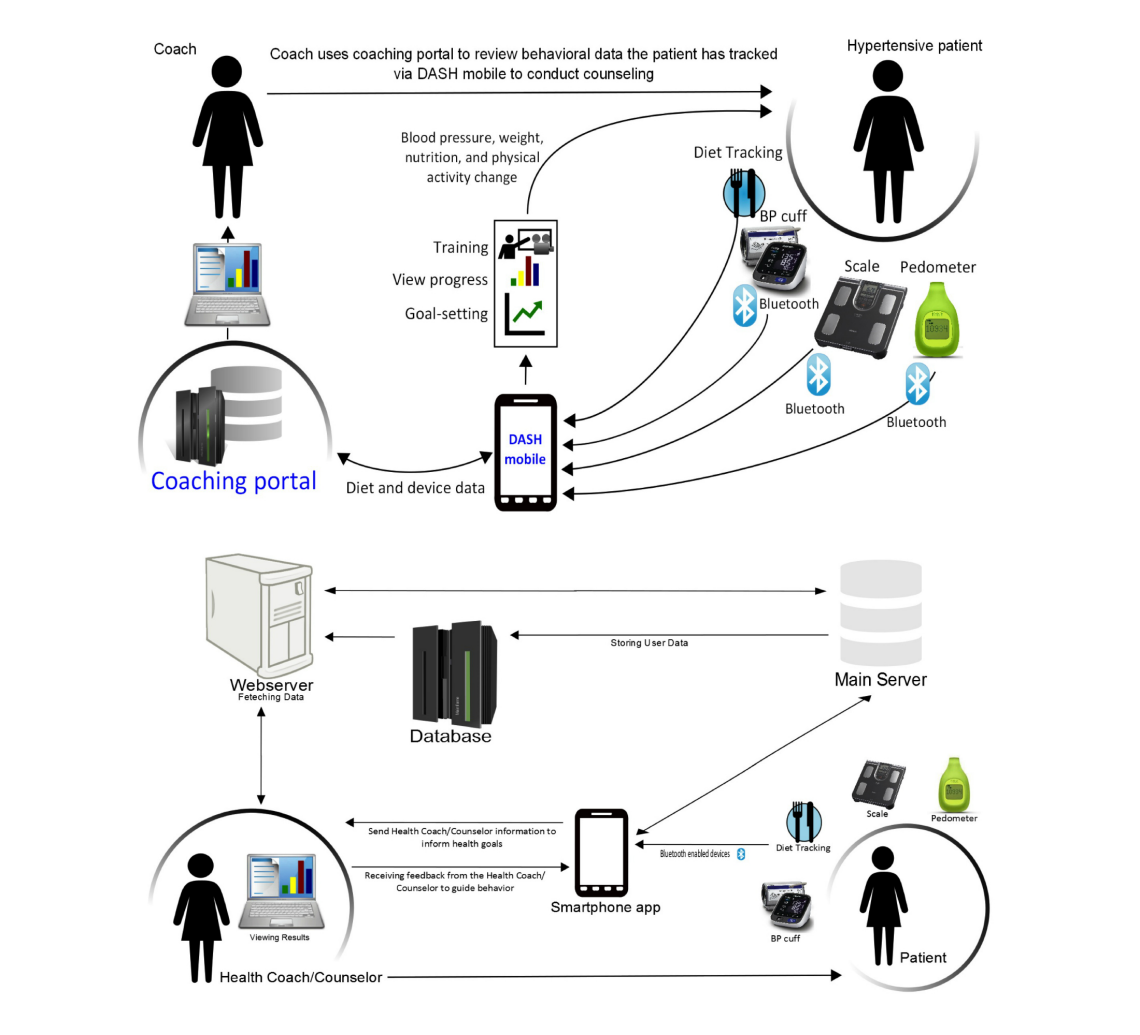
Schematic model of linkages between users, data, and devices of the DASH mobile app.

To minimize barriers to sustainable data entry and avoid draining patient motivation, we made the decision to use wireless device data capture whenever possible. The decision to use a blood pressure monitor, weight scale, and pedometer with wireless communication capabilities included expensive and complicated software development, but we felt strongly that reducing tracking burden was an important principle of sustainable mHealth interventions. In addition, we decided to avoid hardware that required a home Internet connection, as this would exclude patients who use the phone as their primary connection to the Internet. Ultimately, we decided to integrate a Bluetooth blood pressure machine, scale, and pedometer, allowing the patient’s data to be relayed from the device to the phone and then to the Internet (see Figure 3).

#### What Hardware Would a Patient Need to Use the System?

Whether to build the smartphone portion of the system in iPhone or Android operating systems was another early decision. We decided to pursue both for their differing strengths: the iPhone operating system is often more compatible with wireless health devices, yet Android systems are equally if not slightly more popular than iPhone systems [7]. At present, we require that patients own a functional Android or iPhone smartphone with a data plan, as we believe to provide either of these components would represent an unrealistic delivery model for broad implementation of our system.

#### What Core Behavior Change Techniques Should We Include?

**Figure 4:**
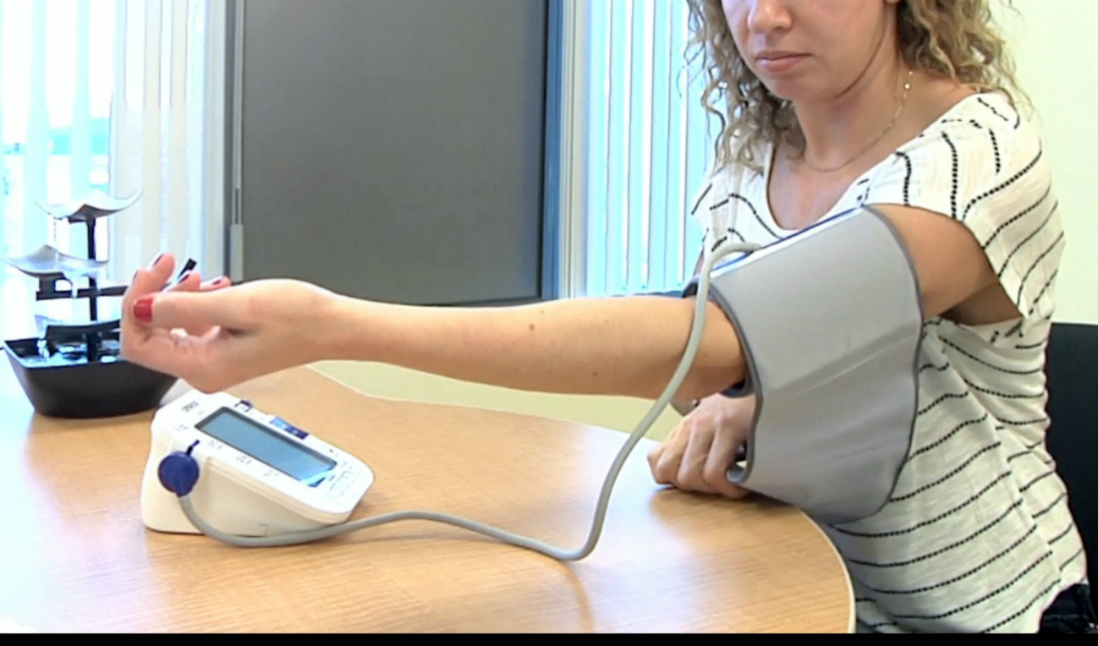
Screenshot from sample training video.

The list of potential behavior change techniques is enormous and the COM-B, Elaboration Likelihood Model, and other frameworks we were employing are robust—supporting the implementation of many specific behavioral tools [25,26]. In order to achieve our “minimally viable product”, we needed to focus on a small core set of functionalities. Cross-referencing the best evidence with the realities of mHealth and behavioral hypertension management, we selected the following core features: (1) automated tracking and feedback, (2) multimedia training and educational clips, and (3) mHealth coach synchronous and asynchronous communication based on principles of motivational interviewing. The wireless devices would provide the automated tracking of weight, physical activity, and blood pressure, while feedback with DASH diet adherence would be acquired through self-report using a simple smartphone interface (Figure 2). The goal setting would be negotiated via mHealth coach communication and tracked via the smartphone. The multimedia training and educational content would be a combination of short videos (Figure 4) and short slide sets (≤2 minutes). We developed original content as well as sourced content from external educational reference materials. The mHealth coaching would be delivered using a motivational interviewing-based guide (manuscript under review), with the synchronous communication occurring via FaceTime, Skype, or Google Hangout video calls when feasible or telephone. The guide is meant to be highly structured, providing section-by-section scripted content with examples of wording and questions, but flexible enough to allow the counselor to follow up on participants’ answers to allow for a natural conversation and to build rapport. Synchronous communications capitalize on the inherent capabilities of a human coach who explores important motivational interviewing-based concepts (eg*,* level of self-efficacy and importance in changing behavior), elucidate relevant multilevel factors that may influence behavior (eg, time, money, community), and conduct collaborative goal setting targeted to the participant’s level of motivation. Synchronous coaching sessions are alternated with shorter asynchronous sessions to review progress, discuss new goal attainment strategies as needed, and provide additional tailored education if requested by the participant. Overall, we sought to strike a balance between the more time-consuming synchronous sessions when the e-coach discusses new behaviors and less time consuming asynchronous sessions checking in about behavioral progress.

### Building an Initial Prototype

While there are established procedures for design specifications in health technology [27,28], in this small-scale health development project, a simplified approach in which we enumerated system capabilities and used them to drive the initial prototyping was a useful starting point. Using the functionality requirement decisions described above, the prototype development began by identifying “use case scenarios”, which are detailed descriptions of how the system will be used in various settings and whether the design specifications are different under various clinical conditions. For example, one use case scenario would describe how the app will function if the patient is expected to access it daily, weekly, etc. Then, employing rapid iterative design methods we create mock-ups of the user interface [29]. Mock-ups allow visualization of how a user would interact with the components of the app. In the UI wireframes, we were able to compare various strategies for tracking dietary intake, visualizing blood pressure and weight data, setting up libraries of relevant information and links, framing the self-management training tutorials, and plan more advanced functions for future versions of our system like motivational alarms, social networking, and GPS-based alerting. In the simulated UI environment, the study team quickly mocked up what various ideas might look like and evaluated their usability on the smartphone interface. We then elicited additional usability feedback from 3 test users who reflected the ultimate target patient population (hypertensives with smartphone access). While a complete description of this user-centered design process is beyond this scope of this paper, some examples of design considerations driven by the test users included (1) lack of clear feedback that their device data was being received and processed (eg, “No way of knowing whether the reading went through”), (2) UI improvements, for example, the addition of 1/2 portion sizes, (3) integration of serving size references directly into the tracking tool icons (eg, “I’m always referring to the website on the education tab to figure out my serving sizes”), and (4) new features such as tracking water and sleep. This feedback and subsequent design modifications encountered during the iterative UI design phase were critical to enhancing development efficiency, usability, and usefulness of the DASH mobile system.

### Development of the Systems Architecture

A flexible, scalable, and integrated “back end” is equally important to an evidence-infused “front end” of any mHealth system. The seamless integration of the UI, mHealth coaching portal, sensing devices, and multimedia content all depend on an underlying architecture to support the system functionality. All of our “back-end” decisions were guided by the following six mHealth platform criteria: (1) a generic database, (2) a scalable Web server, (3) integrated smartphone data collection tools (ie, Bluetooth), (4) a stable server for receiving data, (5) support for big data analytics, and (6) security considerations.

An interactive platform between coach and patient is needed. However, a single system that allows direct communication between coach and patient does not exist. To address this issue and respect the above criteria, we created a Web server to access patient data, process requests, communicate with a patient, display patient data, store patient information, and analyze patterns in a central database. We developed a smartphone application that could interact and provide feedback to the user without storing data locally on the phone or the app to maximize data security. To further support data security, only encrypted and nameless data would be transmitted to the mobile app. To tie these systems together, we built a central server that communicates with the app and collects data for storage, from which the Web server can retrieve and run data analysis.

Figuring out how the data will move through the system—from phone, to server and back again—and the respective security considerations is an active area of research in mHealth [30,31]. All data visualizations on the phone accessed from the server are not locally stored on the smartphone. In addition, considerations regarding what personally identifiable information are linked to the study data needs to be taken into account. Figure 3 represents the final “back-end” architecture of DASH mobile that demonstrates each of these principles and decisions.

## Results

### Lessons Learned

Imagine now that our primary care patient Dawn comes back for her 3-month follow-up appointment. Both she and her provider have forgotten about the DASH diet brochure. Fortunately, in the meantime, the provider’s practice has gained access to the DASH mobile system. The provider e-prescribes the app to Dawn through the electronic medical record, which triggers an automated mailing of the integrated Bluetooth scale, blood pressure cuff, and pedometer to Dawn’s home and a secure message to her (via a patient portal in the electronic health record) with a hyperlink to download the app. Dawn receives the package the next day, downloads the app to her own personal smartphone, and begins the set-up process. Within minutes, Dawn begins watching videos on her phone to learn how to use the system and is contacted that week by the practice health coach to arrange a video call to discuss expectation and goals of the system and arrange future counseling sessions over the coming months.

Over the past 12 months, our study team has faced many challenges to developing this future experience for Dawn, and we have learned are the following 10 lessons. (1) To truly leverage mHealth, you need to be willing to break from tradition and re-imagine behavior change tools in a connected environment, from the outset. At the same time, not everything is done better via mHealth. Each decision, each functionality, and each device need to be evaluated through the multidisciplinary team lens and justified as to why it needs to be rebuilt in an mHealth approach. (2) Development teams need broad membership—crossing several disciplines—informatics, computer science, behavioral science, and clinical medicine. Each group needs equal footing throughout the design process to ensure a product that is robust to the variation in real world implementations. Giving presentations at academic conferences within and outside one’s discipline also helps to inform intervention development by inviting a range of multidisciplinary perspectives. (3) Behavior change frameworks such as COM-B, Elaboration Likelihood Model, and others can be useful scaffolds for organizing the design specifications and potential mHealth behavior change tools; however, developers should be ready to be responsive to new frameworks developed specifically for mHealth interventions as they emerge [32]. (4) Clearly identifying the scope of the initial prototype and matching it to the design specifications ensures efficient development timelines and allows for well-defined iteration cycles. (5) Involving humans such as coaches in mHealth systems is still often critical to a robust intervention, but the pros and cons of doing so need to be carefully evaluated. (6) Decisions around supported platforms, human and technology resources, and workflows should be explicit as they dramatically affect potential future scalability and dissemination potential. The target population, their characteristics, resources, and capabilities should be kept in mind when making these decisions. (7) While the behavior change literature and frameworks enumerate many possible tools, prototypes should focus on evidence-based core tools first in order to ensure rapid development of a minimally viable product and allow for evidence-guided iteration cycles. (8) It is important to keep the prototyping lightweight and simple, particularly in academic environments where resources are often thin. This allows for quick assessments and alterations of the developer’s execution of the design specifications. (9) Whenever possible, it is best to build the prototype in a flexible, scalable fashion as there can be many pivots and evolutions of the system as the development process unfolds. Having a flexible architecture allows for adoption of new data inputs, new devices, and new workflows without massive redesign of the system. This also allows for maximum adaptability for taking on new behaviors or health conditions as opportunities arise (eg, emerging clinical needs, new funding announcements). (10) Engaging with the technology transfer/commercialization experts at your institution early allows you to prepare for inevitable conversations with potential industry partners and for identifying growing intellectual property and inventorship rights.

## Discussion

### Future Plans

This case study describes our experience developing a behavioral blood pressure reduction mHealth system designed to leverage the unique capabilities of smartphones and wireless sensors to facilitate learning, utilization, engagement, and motivation. Our DASH-inspired prototype is focused on initial functionality, content delivery, tracking, and communication with the e-coach. Using this minimally viable product, we are now engaged in usability studies to collect more data about the usability and usefulness of each component of the system and will use these data to improve the app through iterative development [33,34].

Our project is entering its first series of pilot trials, which are a critical first test of its feasibility and preliminary efficacy. Unlike commercial health apps and systems, an mHealth system originating from the medical community requires validation of its clinical efficacy prior to widespread implementation and dissemination. We will conduct short pilots with flexible methodology that allows continued iterative improvement. In addition, we have created an mHealth behavior change architecture that serves as a platform for other common behavior change sensitive conditions such as medication adherence, cancer prevention and control, and diabetes.

### Conclusions

mHealth represents an important turning point in behavioral change interventions. The availability of a context-sensitive, wireless sensor–integrated delivery platform that is literally in the hand of the patient nearly all the time represents a potentially important disruptive force. Researchers need to carefully integrate behavior change best practices, mHealth technologies, and flexible, learning design processes to maximally leverage this potential and substantially change our ability to improve our patients’ behaviors for hypertension and other common chronic conditions.

## Abbreviations

DASH: Dietary Approaches to Stop Hypertension
UI: user interface
